# *Homburgvirus* LP-018 Has a Unique Ability to Infect Phage-Resistant *Listeria monocytogenes*

**DOI:** 10.3390/v11121166

**Published:** 2019-12-17

**Authors:** Yaxiong Song, Tracey L. Peters, Daniel W. Bryan, Lauren K. Hudson, Thomas G. Denes

**Affiliations:** Department of Food Science, University of Tennessee, Knoxville, TN 37996, USA; ysong35@vols.utk.edu (Y.S.); tpeter21@vols.utk.edu (T.L.P.); dbryan8@utk.edu (D.W.B.); lkhudson@utk.edu (L.K.H.)

**Keywords:** bacteriophage, phage, *Listeria monocytogenes*, phage resistance, *Homburgvirus*, variant mapping, complete genome

## Abstract

*Listeria* phage LP-018 is the only phage from a diverse collection of 120 phages able to form plaques on a phage-resistant *Listeria monocytogenes* strain lacking rhamnose in its cell wall teichoic acids. The aim of this study was to characterize phage LP-018 and to identify what types of mutations can confer resistance to LP-018. Whole genome sequencing and transmission electron microscopy revealed LP-018 to be a member of the *Homburgvirus* genus. One-step-growth curve analysis of LP-018 revealed an eclipse period of ~60–90 min and a burst size of ~2 PFU per infected cell. Despite slow growth and small burst size, LP-018 can inhibit the growth of *Listeria monocytogenes* at a high multiplicity of infection. Ten distinct LP-018-resistant mutants were isolated from infected *Listeria monocytogenes* 10403S and characterized by whole genome sequencing. In each mutant, a single mutation was identified in either the LMRG_00278 or LMRG_01613 encoding genes. Interesting, LP-018 was able to bind to a representative phage-resistant mutant with a mutation in each gene, suggesting these mutations confer resistance through a mechanism independent of adsorption inhibition. Despite forming plaques on the rhamnose deficient 10403S mutant, LP-018 showed reduced binding efficiency, and we did not observe inhibition of the strain under the conditions tested. Two mutants of LP-018 were also isolated and characterized, one with a single SNP in a gene encoding a BppU domain protein that likely alters its host range. LP-018 is shown to be a unique *Listeria* phage that, with additional evaluation, may be useful in biocontrol applications that aim to reduce the emergence of phage resistance.

## 1. Introduction

*Listeria monocytogenes* is a Gram-positive, opportunistic, foodborne pathogen that has the capacity to cause the potentially fatal disease listeriosis [1,2]. *Listeria monocytogenes* is ubiquitous in the environment, where it can survive and grow over a wide range of environmental conditions [3,4,5]. This makes it a particularly problematic pathogen to control in the food and food processing environment, as it can grow at refrigeration temperatures and under low pH and high salt conditions [6,7]. It is estimated that there are ~1600 cases of human listeriosis per year in the United States, with a ~19% mortality rate [8]. Costs associated with these cases are estimated to exceed $2.8 billion in economic losses [8]. Due to the high mortality rate and large financial burden caused by listeriosis, a “zero tolerance” policy was adopted in 1985 by the U.S. Food and Drug Administration for the detection of *L. monocytogenes* in ready-to-eat foods [9]. Listeriosis is also a global problem, with an estimated total of 23,150 cases in 2010 [10]. In 2017, South Africa recalled $52.9 million worth of polony (a processed meat product) due to the largest confirmed listeriosis outbreak to date. There was a total of 1060 confirmed cases and 216 known deaths associated with the outbreak [11].

Bacteriophages, or “phages”, have been used in biocontrol products targeting *Listeria monocytogenes* for over a decade [12]. A couple of the key advantages of using phages to control *L. monocytogenes* are that they are highly host-specific and can self-replicate wherever contamination by the host bacteria is encountered [13,14]. However, one of the key challenges limiting the potential long-term efficacy of phage-based biocontrols is the emergence of phage resistance in the treated environment. One study showed that phage-resistant mutants of *L. monocytogenes* were selected from 95 out of 110 phage-infected cultures [15]. Another study showed that phage-resistant strains were isolated from specific Austrian dairies only after phage biocontrol products were used in the dairies [16]. Previously characterized phage-resistant mutants have been shown to confer resistance through adsorption inhibition due to loss of phage receptor sugar moieties associated with the cell wall teichoic acids [15,17,18,19]. Recently, Trudelle et al. showed that phage resistance in serotype 1/2a strains, caused by loss of rhamnose in the wall teichoic acids, demonstrated resistance against 119/120 phages from a diverse phage collection. The authors of this study concluded that this type of mutation may represent a serious challenge for phage-based biocontrol [18]. In this study we characterize *Listeria* phage LP-018, the only phage capable of forming plaques on the phage-resistant mutant lacking rhamnose in its cell wall teichoic acids, and the phage-resistant mutants that are selected by this unique phage.

## 2. Materials and Methods

### 2.1. Bacterial Strains and Bacteriophages

*L. monocytogenes* strain MACK was used for phage enumeration and amplification (Table 1 and Supplemental Table S1). *L. monocytogenes* strain 10403S and 10403S-derived mutants were the model strains used in the reported experiments. FSL D4-0014, referred to here as “10403S (GlcNAc^−^)” and FSL D4-0119, referred to here as “10403S (Rha^−^)” are previously characterized phage-resistant mutants of 10403S [15]. UTK S1-0004 and UTK S1-0010 are 10403S-derived mutants isolated in this study that resist LP-018 infection (Table 1), referred to in this paper as “10403S (m_acid-resistance)” and “10403S (m_foldase)”, respectively. All *Listeria monocytogenes* strains were stored at −80 °C in Brain Heart Infusion (BHI) with 15% (wt/vol) glycerol and grown at 37 °C on 1.5% (wt/vol) BHI agar plates. Liquid cultures of *L. monocytogenes* were started by inoculating BHI broth with a single colony from a streak plate and incubating at 37 °C with shaking at 160 RPM.

*Listeria* phages LP-048 and LP-125 are well-studied phages in the *Pecentumvirus* genus that are not able to infect mutant strains of *Listeria monocytogenes* lacking rhamnose in their cell wall teichoic acids [15,18]. *Listeria* phage LP-018 was the only phage found to form visible plaques on 10403S (Rha^−^) [18]. Phage titers were enumerated on lysogeny broth morpholino-propane sulfonic acid (LB-MOPS) agar supplemented with 0.1% glucose, 1 mM CaCl_2_, and 1 mM MgCl_2_ by top agar double-layer overlay, and were incubated at 25 °C overnight (16 ± 2h).

### 2.2. Morphological Observation of LP-018 by Transmission Electron Microscopy

High titer stocks of LP-018 were centrifuged at 21,000× *g* for 60 min, and the pellet was washed with 0.1 M ammonium acetate solution (pH 7). The washing step was repeated twice, then LP-018 samples were applied to 200-mesh carbon-coated copper grids and stained with 1% phosphotungstic acid (PTA; pH adjusted to 7.4 with NaOH). After staining, the samples of LP-018 were imaged on a JEOL JEM-1400 TEM (JEOL, Inc., Peabody, MA, USA) at 80kV. Morphological characterization was performed with the Gatan Microscopy Suite Software (version 3; Gatan, Pleasanton, CA, USA) and analyzed with ImageJ (version 2.0.0-rc-69/1.52).

### 2.3. Isolation of Phage-Resistant Mutants

To isolate *Listeria monocytogenes* 10403S mutants resistant to LP-018, two different methods were used. The first method was to combine 100 μL of a high titer stock (3.3 × 10^10^ PFU/mL) with 30 μL of an overnight culture of 10403S and 3 mL of molten LB-MOPS top agar, gently vortexed, then poured onto LB-MOPS plates. The second method was to pipette 100 μL of a high titer LP-018 stock (3.3 × 10^10^ PFU/mL) on to a top agar lawn of 10403S and gently spread the phage with a sterile cell spreader to evenly distribute the phage. All plates for both methods were incubated at 25 °C overnight, and individual colonies were selected as mutants resistant to phage LP-018. All isolated resistant mutants were tested with LP-018 by spot assay to confirm phage resistance.

### 2.4. DNA Extraction and Genomic Analysis

An adapted version of the Extraction of Bacteriophage λ DNA from Large-scale Cultures Using Formamide protocol by Sambrook and Russel was used [24]. High titer phage stocks (>5 × 10^10^ PFU/mL) were pretreated with 2 mM CaCl_2_, 5 μg/mL DNase I (Promega BioScience, Madision, WI, USA), 30 μg/mL RNase A (Sigma-Aldrich, St. Louis, MO, USA), and incubated at room temperature for 30 min to remove exogenous genomic material. Samples were incubated at 65 °C for 10 min to inactive enzymes before adding 2 mg/mL Proteinase K, following the rest of the Sambrook and Russel protocol. Gel phase-lock tubes (light gel, QuantaBio cat# 10847-800) were used for phase separation. DNA pellets were resuspended in 10 mM Tris-buffer (pH 8.0). The concentration and quality of the extracted DNA were measured on a Nanodrop One spectrophotometer (ThermoFisher Scientific Inc., Waltham, MA, USA). DNA of LP-018-resistant *Listeria* was extracted with the Qiagen DNA Easy mini kit (Qiagen GmbH, Hilden, Germany) with modifications to the manufacturer protocol, as previously described [15].

Sequencing libraries were prepared using NexteraXT library kits (Illumina, San Diego, CA, USA), and sequenced using an Illumina MiSeq v.3 instrument, with 300 paired-end read chemistry and 275 cycles, or an Oxford Nanopore Technologies MinION. Raw Illumina reads were trimmed using Trimmomatic V0.35 [25] and checked for quality using FastQC v0.11.7 [26]. For variant analysis of LP-018-resistant bacterial mutants, trimmed reads were run in McCortex v.0.0.3 against the 10403S RefSeq assembly (RefSeq ID 376088) with joint calling and kmer size of 57 [27]. VCF output files were annotated using SnpEff v4.3t [28]. For phage genome assemblies, reads were assembled into single contigs using the hybrid assembler, Unicycler [29], or SPAdes v3.12.0 [30]. Assembly statistics were generated using BBMap v38.08 [31], SAMtools v0.1.8 [32], and Quast v4.6.3 [33]. The LP-018 genome was annotated using RASTtk using the customized pipeline (“annotate-proteins-phage” moved above “annotate-proteins-kmer-v2”) [34]. The annotation was manually inspected and updated using InterProScan [35]. JSpeciesWS was used to analysis the relatedness of LP-018 with other P70-like (*Homburgvirus*) phages using the average nucleotide identity MUMer (ANIm) method [36]. Sequencing data can be accessed from NCBI bioproject accessions PRJNA544490 and PRJNA544516.

### 2.5. One-Step-Growth Experiment

To determine the growth characteristics of LP-018 in liquid media, a one-step growth experiment was performed using an adapted version of the protocol described in Denes et al. [15]. We added 500 μL of the overnight culture of *Listeria monocytogenes* 10403S into 50 mL LB-MOPS with 0.1% glucose, 1 mM CaCl_2_, and 1 mM MgCl_2_ in a 250 mL flask. The culture was incubated in a shaking water bath at 25 °C and 160 RPM. When the optical density at 600 nm (OD_600_) grew to 0.1, 1 × 10^9^ PFU of LP-018 was added to the culture (multiplicity of infection (MOI) of ~0.1). For each time point, two samples were taken. To measure infected host cells and unabsorbed viable phages, one sample was immediately diluted with phosphate-buffered saline (PBS) and enumerated by the double-layered agar method. To measure the total concentration of viable phages, the other sample was transferred into a dilution tube with 50 μL chloroform and gently mixed, then after at least 15 min, the aqueous phase was diluted and enumerated by the double-layered agar method. To prevent the lysis of infected cells due to shear forces from micropipetting during sample collection and dilution, 60 min after infection, the infected culture was portioned and diluted in 15 mL centrifuge tubes with 9 mL of fresh media using serological pipettes to three concentrations appropriate for plating without additional dilution. This dilution also served to limit further adsorption of LP-018, which would impact the synchronicity of the infection. After being portioned and diluted, samples were plated directly from the diluted tubes using 1 mL micropipettes. After plating, 5 mL of each diluted infection was transferred to a 250 mL flask containing 45 mL of fresh media. These flasks were then incubated at 25 °C with shaking at 160 RPM. All further samples were plated directly from these flasks using 1 mL micropipettes.

### 2.6. Growth Inhibition Assay of Listeria monocytogenes by LP-018

Experimental cultures were started by inoculating 5 mL LB-MOPS supplemented with 0.1% glucose, 1 mM CaCl_2_, and 1 mM MgCl_2_ with 50 μL of overnight culture. The experimental cultures were then incubated at 25 °C with shaking at 160 RPM. At an OD_600_ of 0.1, each culture was diluted 100-fold in fresh supplemented LB-MOPS, and each bacterial strain was transferred into one 15 mL tube for each experimental condition tested. Each tube was then infected with LP-018 at the target MOI or with SM buffer (control). The OD_600_ of each infection was measured every 0.5 h for 12 h. The experiment was replicated three times for each experimental condition.

### 2.7. Phage Adsorption Assay

*L. monocytogenes* cultures were grown to an OD_600_ of 0.1. Each culture was then portioned into three 1 mL aliquots and infected with 20 μL of LP-018, LP-048, or LP-125 at 1 × 10^9^ PFU/mL. The infected aliquots were incubated at 25 °C with shaking at 160 RPM. After 15 min for the aliquots containing LP-048 or LP-125, and after 80 min for the aliquot containing LP-018, the tubes were centrifuged for 1 min at 17,000× *g* and 4 °C. The supernatant was then immediately diluted in PBS and enumerated by the double-layer agar method. The experiment was replicated three times and values reported for each experiment were the average of two duplicate samples (technical replicates). Percent binding values were analyzed using JMP Pro (Version 14.0.0; SAS Institute Inc., Cary, NC, USA). A linear model was constructed using the factors *L. monocytogenes* strain, phage, *L. monocytogenes* strain*phage (cross), and biological replicate (random factor), with percent binding as the model response. Pairwise comparisons were made using Tukey’s honestly significant difference (HSD) test. The significance level was set at *p* < 0.05.

## 3. Results and Discussion

### 3.1. Characterization of LP-018

#### 3.1.1. LP-018 Shares Morphology with Phages of the Genus *Homburgvirus*

**Figure 1:**
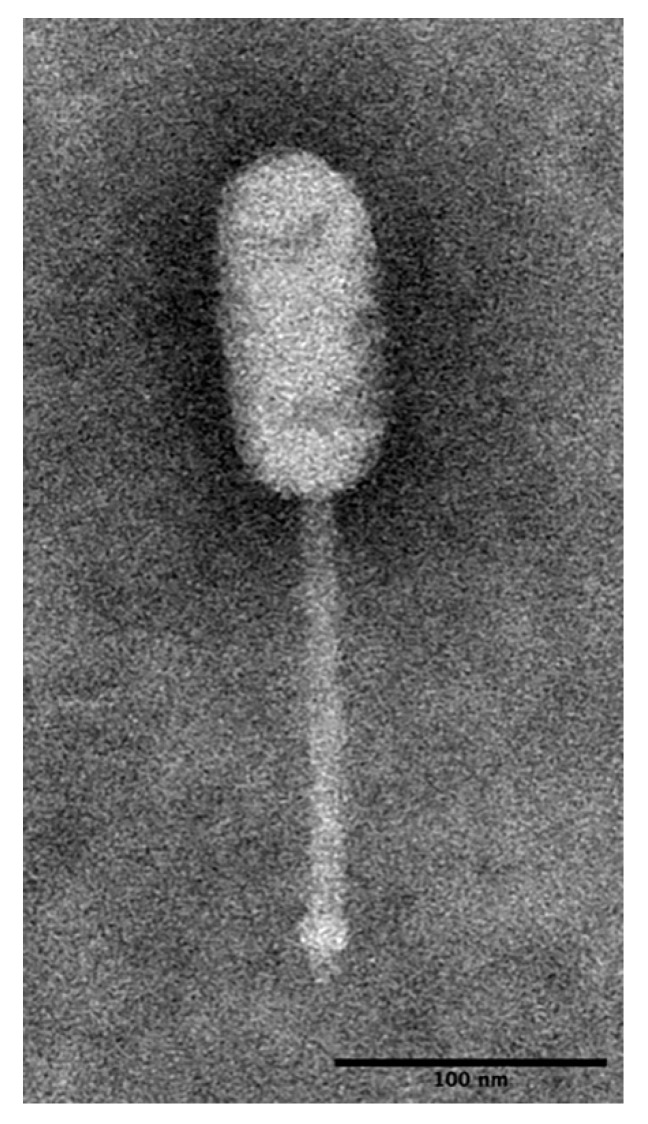
Transmission electron micrograph of phage LP-018. The sample was stained with 1% phosphotungstic acid (PTA) at pH 7.4 and imaged at 80 kv with a final magnification of 41,000×.

Based on the observed morphological characteristics (Figure 1), LP-018 was identified as a likely member of the genus *Homburgvirus*, belonging to the family *Siphoviridae* in the order of *Caudovirales*. LP-018 had an elongated capsid (63.2 ± 4.67 nm × 132.2 ± 3.68 nm, *n* = 33) with a non-contractile tail (168.02 ± 9.15 nm in length). This morphology is nearly identical to other homburgviruses [23,37,38]. Interestingly, the morphology is also shared by *Enterococcus* phage VD13 [39], which was previously shown to cluster by genomic analysis with sequenced homburgviruses [23].

#### 3.1.2. Whole Genome Sequencing Confirms LP-018 is a *Homburgvirus*

**Figure 2:**
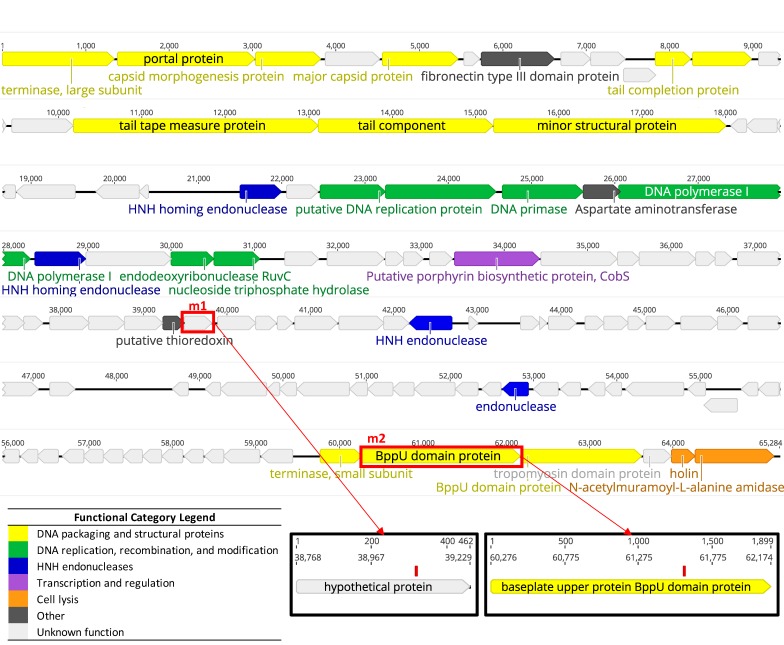
Genome map of LP-018 showing predicted coding sequences. Gene colors correspond to functional category: DNA packaging and structural proteins are yellow; DNA replication, recombination, and modification are green; His-Asn-His (HNH) endonucleases are blue; transcription and regulation are purple; cell lysis are orange; others are dark gray; and unknown (including hypothetical proteins and phage proteins (annotations not shown)) are light gray. Coding sequences containing mutations identified in this study are in red boxes. The figure was made with Geneious Prime (v. 2019.1.1).

The *Listeria* phage LP-018 genome assembled into a single contig of 65.3 Kbp that had an average read coverage of 823. The G + C content of the genome was 36.4%. It contained 112 predicted coding sequences, of which 85 were annotated as hypothetical proteins or phage proteins without known specific functions (Figure 2). The genome contained no tRNAs. As the morphological characteristics of LP-018 match the features of homburgviruses, the LP-018 genome nucleotide identity was compared with all other sequenced homburgviruses. The results show that LP-018 was most closely related to LP-037, with an ANIm of 97.71% across 96.74% of its aligned nucleotide sequence (Table 2). As the total genome sequence identity between LP-018 and LP-037 was 94.52%, which is lower than 95%, we propose that LP-018 be classified as a new species belonging to the genus *Homburgvirus* [40].

### 3.2. Growth Characteristics of LP-018

#### 3.2.1. One-Step-Growth Experiment

**Figure 3:**
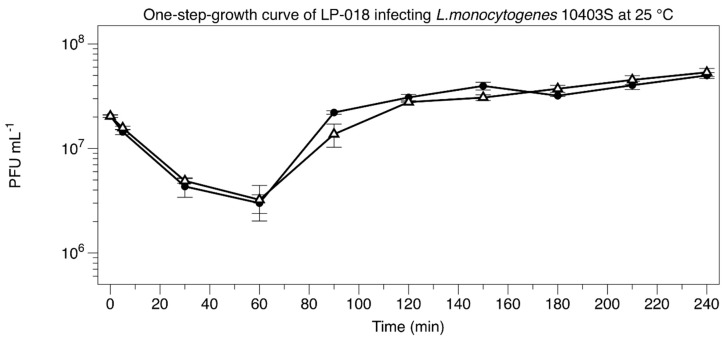
Low multiplicity of infection of 10403S with LP-018 at 25 °C. Closed circles represent the phage titer in chloroform treated samples; open triangles represent the phage titer in untreated samples. Data are mean values of three biological replicates, and error bars represent standard error.

LP-018 was found to have a significantly longer adsorption time and a much smaller burst size than either LP-048 and LP-125; the only other *Listeria* phages we know to have published one-step growth curve data [15]. At 60 min post-infection, 85.1% (5.63% standard deviation) of LP-018 had adsorbed to *L. monocytogenes* 10403S (Figure 3). As a comparison, 78.2% of LP-048 and 99% of LP-125 adsorbed to 10403S in 20 min [15]. The latent period of LP-018 could not be determined, as infected cells without viable internal phages were below the limit of detection. A possible explanation for this is that cells newly infected by LP-018 could be extremely fragile and are thus lysed during the dilution or plating of the unchloroformed samples. In a typical one-step growth experiment, we would have expected the unchloroformed samples to remain stable at the beginning of the experiment [23]. The eclipse period of LP-018 was between 60 min and 90 min, which is much longer than the *Pecentumvirus* phages previously tested; the eclipse period of LP-048 and LP-125 were only 40–50 min and 35–40 min, respectively [15]. The calculated burst size of LP-018 was approximately 2 PFU/cell, which was much lower than LP-048 (13.6) and LP-125 (21.3); however, the slow adsorption rate made calculating a burst size that represents the true burst size extremely challenging. Interestingly, LP-018 appears to perform differently in liquid media and solid media. LP-018 appears to produce clear, well-defined plaques on solid media, but in liquid media (under the conditions tested) shows unexpectedly slow binding and small bursts after a relatively long infection period.

#### 3.2.2. Growth Inhibition of *Listeria monocytogenes* by LP-018

**Figure 4:**
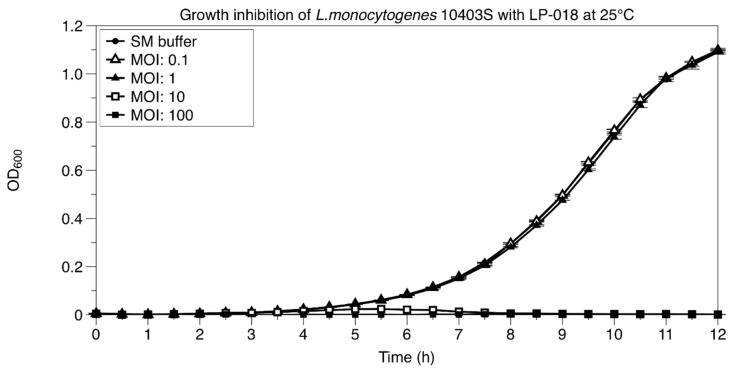
Bacterial growth of 10403S treated with different concentrations of LP-018. Closed circles represent the uninfected control (SM buffer), open triangles represent the infection condition at a multiplicity of infection (MOI) of 0.1, closed triangles represent the infection condition at a MOI of one, open squares represent the infection condition at a MOI of 10, closed squares represent the infection condition at an MOI of 100. The SM Buffer control and infection conditions at MOI of ≤1 were indistinguishable. Data are mean values of three biological replicates, and error bars represent standard error.

The growth of 10403S treated with different concentrations of LP-018 was determined (Figure 4). The results showed that LP-018 was not capable of inhibiting the growth of 10403S at a multiplicity of infection (MOI) of one or lower. However, at higher MOI’s (10 and 100), LP-018 could notedly keep the OD_600_ of *L. monocytogenes* 10403S under 0.1 for 12 h. Lower concentrations of LP-018 (MOI’s ≤ 1) had no observable effect on the growth of 10403S, possibly due to its slow adsorption rate and small burst size.

### 3.3. Isolation and Characterization of LP-018-Resistant Mutants

#### 3.3.1. Isolation of LP-018-Resistant Mutants

Characterization of phage-resistant mutants can be a useful tool for identifying potential phage receptors [15,41]. It is also an important step in designing cocktails specifically to reduce the frequency of phage resistance selection. In this study, we were able to isolate 10 distinct phage-resistant mutants of 10403S by LP-018. We selected one mutant from each of the 10 selection experiments (i.e., we successfully isolated a phage-resistant mutant from each attempt). All isolated mutants were confirmed to be resistant to LP-018 infection by spot assay.

#### 3.3.2. Genetic Characterization of LP-018-Resistant Mutants

**Figure 5:**
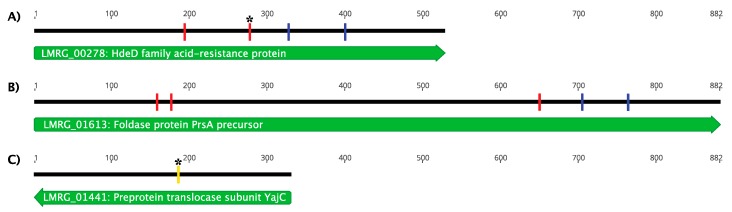
Map of mutations identified in mutant 10403S strains resistant to LP-018. Mutations were identified in (**A**) LMRG_00278, an HdeD family acid-resistance protein, (**B**) LMRG_01613, a foldase protein PrsA precursor, and in (**C**) LMRG_01441, a preprotein translocase subunit YajC. In nine of the ten phage-resistant mutants, only one mutation was present, while one phage-resistant mutant had two mutations in two different genes (designated by asterisks). The numbers in the figure represent the nucleotide position of the coding sequence in each gene. Nonsense mutations are designated by red marks, frameshift mutations are designated by blue marks, and missense mutations are designated by yellow marks.

Whole-genome sequencing of LP-018-resistant mutants revealed that all identified mutations mapped to three genes on the 10403S chromosome. Of the phage-resistant mutants, nine were found to contain only a single mutation. Three of these mutations were found in LMRG_00278, which was annotated as an HdeD family acid-resistance protein, and six of these mutations were found in LMRG_01613, which was annotated as a precursor to the foldase protein PrsA2 (Figure 5). Expression of *LMRG_00278*, which encodes the putative HdeD family acid-resistance protein, an uncharacterized membrane protein, is known to be regulated by the general stress response alternative sigma factor B [42,43]. If LP-018 infection depends on the expression of *LMRG_00278*, further work should be conducted to determine if LP-018 shows improved lytic activity against *L. monocytogenes* under σB active conditions [44]. Interestingly, PrsA2 is a post-translocation chaperone that contributes to the virulence of *L. monocytogenes* by stabilizing and promoting the activity of secreted virulence factors [45,46,47]. This suggests that LP-018 may select for phage-resistant mutants that are less virulent than the parental strain. Identifying mutants in these genes was a surprising result as all previously characterized phage-resistant mutants have been identified in genes affecting cell wall teichoic acids, and were found to confer resistance by inhibiting adsorption [15,17,18,19]. One of the ten phage-resistant mutants was found to have two mutations: one nonsense mutation in *LMRG_00278* (total of four phage-resistant mutants had an identified mutation in this gene) and a missense mutation in *LMRG_01441*, which was annotated as encoding a preprotein translocase subunit of YajC. The missense mutation in *LMRG_01441* resulted in one amino acid change from a glycine to a cysteine with no other notable predicted effects on the protein product. For this reason, and because all other mutations occurred in genes *LMRG_00278* and *LMRG_01613*, the characterization of this gene was not investigated further. We selected a representative phage-resistant strain with a mutation in either gene *LMRG_00278* or *LMRG_01613* for further characterization. We selected the representative mutants based on our prediction of which mutants would be expected to have the greatest effect on the function of the target gene. UTK S1-0010, referred to throughout the remainder of this article as 10403S (m_acid-resistance), was selected as it was the mutant strain with a nonsense mutation at nucleotide position 160/882, and was predicted to result in the expression of a truncated protein (54 /294 amino acids). UTK S1-004, referred to throughout the remainder of this article as 10403S (m_foldase), was selected as it is the mutant strain with a nonsense mutation at nucleotide position 65/176 and is predicted to result in the expression of a truncated protein (194/ 528 amino acids).

#### 3.3.3. Adsorption Assay with Phage-Resistant Mutants

**Figure 6:**
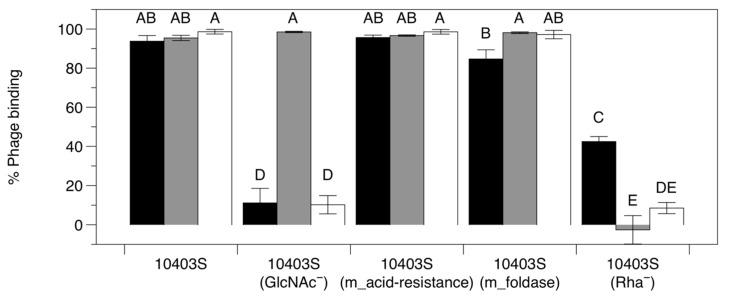
Phage binding of LP-018, LP-048, and LP125 to 10403s and phage-resistant 10403S mutants. Black bars represent LP-018, grey bars LP-048, and white bars LP-125. LP-018 binding was measured after 80 min, and LP-048 and LP-125 binding were measured after 15 min; comparisons between phages should consider these differences. Values are the mean of three biological replicates, and error bars represent standard error. Bars that share the same letter are not significantly different (e.g., bars marked AB are not significantly different from bars marked A or B).

The mutations identified in the LP-018-resistant mutants were found in genes without any obvious effect on the cell’s outer surface. We thus performed adsorption assays to see if we could identify the first phage-resistant mutants in *Listeria* that resist infection through a mechanism independent of adsorption inhibition. Adsorption assays revealed that the mutations in 10403S (m_acid resistance) and 10403S (m_foldase) had no significant impact on adsorption of LP-018 (Figure 6). Interestingly, LP-018 showed significantly reduced binding to the 10403S mutant lacking rhamnose in its cell wall teichoic acids (Rha^−^) and the 10403S mutant lacking N-acetlyglucosamine in its cell wall teichoic acids (GlcNAc^−^); although there was significantly higher binding to the 10403S (Rha^−^) strain than the 10403S (GlcNAc^−^) strain. This was expected, as LP-018 could form clear, distinct plaques on the Rha^−^ strain, which would require some degree of binding capacity for LP-018. This data suggests that (i) N-acetylglucosamine and rhamnose are involved in the adsorption of LP-018 to its host and (ii) resistance conferred by mutations in the acid resistance and foldase genes, *LMRG_00278* and *LMRG_01613*, respectively, is likely through a mechanism independent of phage adsorption inhibition. However, we cannot rule out the possibility that these mutations impact a secondary adsorption step that is dependent on initial adsorption to cell wall teichoic acids. As *LMRG_00278* and *LMRG_01613* encode a putative membrane protein and a secreted foldase, respectively, the mutations observed most likely impact the cellular inner wall zone [48]. Such an effect could potentially block the penetration or translocation steps of phage infection [49]. Growth curves of all *LMRG_00278* and *LMRG_01613* mutants were also performed; no growth defects were observed that could indicate an indirect cause of phage resistance (Supplemental Figure S1).

### 3.4. Growth Inhibition of Phage-Resistant Mutants by LP-018

**Figure 7:**
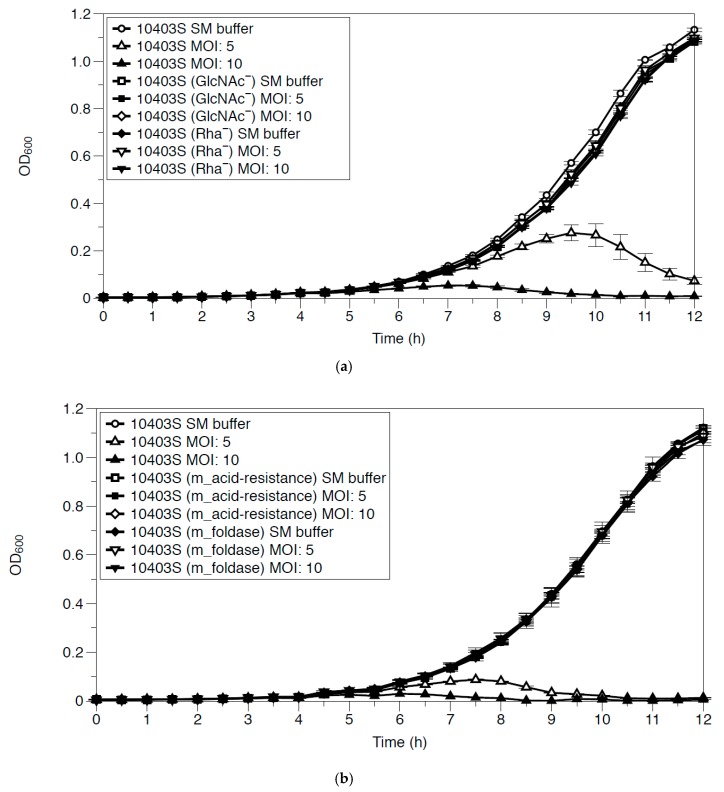
(**a**): Effect of LP-018 on the growth of 10403S (GlcNAc^−^) and 10403S (Rha^−^) at 25 °C. Open circles represent 10403S buffer control, open triangles 10403S infected at MOI five, closed triangles 10403S infected at a MOI of 10, open squares 10403S (GlcNAc^−^) buffer control, closed squares 10403S (GlcNAc^−^) infected at MOI five, open diamonds 10403S (GlcNAc^−^) infected at MOI 10, closed diamonds 10403S (Rha^−^) buffer control, open inverted triangles 10403S (Rha^−^) infected at MOI 5, and closed inverted triangles 10403S (Rha^−^) infected at MOI 10. (**b**): Effect of LP-018 on growth of 10403S (m_acid-resistance) and 10403S (m_foldase). Open circles represent 10403S buffer control, open triangles 10403S infected at MOI five, closed triangles 10403S infected at MOI 10, open squares 10403S (m_acid-resistance) buffer control, closed squares 10403S (m_acid-resistance) infected at MOI five, open diamonds 10403S (m_acid-resistance) infected at MOI 10, closed diamonds 10403S (m_foldase) buffer control, open inverted triangles 10403S (m_foldase) infected at MOI five, and closed inverted triangles 10403S (m_foldaseb) infected at MOI 10. Values are the mean of three biological replicates, and error bars represent standard error.

Due to LP-018’s significantly reduced adsorption efficiency against 10403S (Rha^−^), we performed growth inhibition assays in liquid culture of the 10403S (Rha^−^) and 10403S (GlcNAc^−^) strains. Interestingly, no observable differences were seen between the LP-018 treated and untreated mutant samples (Figure 7a). This suggests that LP-018 is likely not as effective at inhibiting rhamnose deficient phage-resistant mutants in liquid conditions as it is in solid conditions, as was shown by Trudelle et al. [18]. Growth inhibition of the LP-018-resistant mutants 10403S (m_acid resistance) and 10403S (m_foldase) was also tested (Figure 7b). As expected, LP-018 showed no inhibitory effect against these phage-resistant mutants.

### 3.5. Isolation and Characterization of LP-018 Mutants with Different Host Ranges

It was previously observed that LP-018 showed a low level of activity against 10403S (GlcNAc^−^) [18]. A possible explanation for this was that LP-018 had a low-level mutant population responsible for that activity. To test this hypothesis, we plaque purified LP-018 on 10403S (Rha^−^) and 10403S (GlcNAc^−^). The efficiency of plaquing experiments revealed that LP-018_m1 (isolated on the Rha^−^ strain) showed no activity against 10403S (GlcNAc^−^), and LP-018_m2 (isolated on the GlcNAc^−^ strain) showed no activity against 10403S (Rha^−^) (Table 3). This confirmed our hypothesis that there was a subpopulation of LP-018 mutants in the LP-018 stocks. Further, the data suggests that LP-018_m1 requires GlcNAc in the cell wall teichoic acids for binding, and LP-018_m2 requires rhamnose in the cell wall teichoic acids for binding. As expected, LP-018 and LP-018_m1 formed plaques on 10403S (Rha^−^), although at reduced efficiency. The pecentumviruses LP-048 and LP-125 were both able to infect the 10403S (m_acid-resistance) and 10403S (m_foldase) mutants.

Whole genome sequencing of LP-018_m1 and LP-018_m2 was conducted to identify potential mutations. Two were identified (Figure 2). A mutation was present in LP-018_m1 in a gene annotated as a hypothetical protein, and would result in a nonsense mutation leading to a truncated protein product (the stop codon is located at amino acid position 107 of 153). This mutation was located at nucleotide position 39,086 and caused a codon change of GAA to TAA. This mutation was not detected in the sequenced LP-018 sample, and 116/116 reads confirmed the mutation in the LP-018_m1 sample. This suggests the mutation likely occurred in the plaque purification and amplification steps. The host range data collected from this phage mutant does not suggest any change in host range from the dominant phage strain present in the LP-018 stock. The other mutation was present in LP-018_m2 in a gene annotated as a hypothetical protein containing a conserved BppU-family domain and would result in a radical nonsynonymous substitution (the polar and acidic amino acid, aspartic acid would be substituted with the nonpolar valine [50]). This mutation was located at nucleotide position 61,585 and caused a codon change of GAT to GTT. This radical nonsynonymous mutation was detected in the original LP-018 sample at very low levels (1 out of 396 reads supported this variant) and was confirmed to be present in the LP-018_m2 sample by 969/970 of the reads. BppU is a baseplate protein that is part of the host adsorption machinery in Gram-positive-infecting phages [50,51]. This supports the EOP data, suggesting that the mutation was present at very low levels in a subpopulation of LP-018. Further, this data suggests the mutation identified in LP-018_m2 is affects the host range by altering the function of a baseplate protein involved in the adsorption to the host.

### 3.6. Efficiency of Plaquing of Homburgviruses Against LP-018-Resistant Mutants

To test whether the phage-resistant mutants selected for by LP-018 was just specific to LP-018 or to homburgviruses in general, an EOP experiment was performed with four other homburgviruses (*Listeria* phages LP-026, LP-037, LP-110, and LP-114) against 10403S (m_acid-resistance) and 10403S (m_foldase). All four homburgviruses showed no activity against 10403S (m_foldase). Interestingly, *Listeria* phage LP-114 plaqued 89% (18% standard deviation) as efficiently on 10403S (m_acid-resistance) as on 10403S, and LP-026 plaqued 11% (9% standard deviation) as efficiently on 10403S (m_acid-resistance) as on 10403S. This data suggests that these resistance types do broadly effect homburgviruses, but LP-110, and LP0026 to a lesser degree, are able to overcome the resistance caused by a nonsense mutation in the putative acid-resistance protein encoded by *LMRG_00278*.

## 4. Conclusions

LP-018 is a *Homburgvirus* with the unique ability to infect phage-resistant mutants that have lost rhamnose in their cell wall teichoic acids [18]. Sequencing of LP-018 revealed that if genome length is accounted for, it may meet the criteria for qualifying as a new species [40]. Although we did not observe levels of growth that would be characteristic of a potent lytic phage, it is possible that the conditions selected in this study were not ideal for determining the full potential of LP-018. It is known that environmental conditions can have a strong impact on phage infection [44]. For example, Tokman et al. previously showed that growth temperature had a particularly large effect on the plaquing efficiencies of homburgviruses [52]. Future work will be needed to explore if other conditions in liquid media provide more favorable growth of LP-018, and if LP-018 is effective at reducing *L. monocytogenes* concentrations in food matrices.

Most notably, this study identified and characterized the first phage-resistant mutants of *Listeria* that resist phage infection through a mechanism independent of adsorption inhibition. Interestingly, these mutations appear to affect phages in the *Homburgvirus* genus specifically. Further work will need to be conducted to determine the mechanism of resistance conferred through these newly identified mutations. We have also identified a gene in LP-018 that is likely involved in host range determination. Current phage-based products targeting *L. monocytogenes* have all relied on the pecentumviruses of *Listeria* [12]. The work reported here provides knowledge of a unique member of a genus of *Listeria* phage that has not been explored for potential use in food safety applications. Such knowledge of new and diverse phages may be essential for reaching the full potential of phage-based biocontrol while maintain long-term efficacy.

## Figures and Tables

**Table 1:** *Listeria monocytogenes* strains and bacteriophages used in this study.

Strain or Phage	Description	Reference or Original
***Listeria monocytogenes* Strain**		
MACK	Lineage II, Serotype 1/2a	Hodgson, 2000 [20]
10403S	Lineage II, Serotype 1/2a	Bishop and Hinrichs, 1987 [21]
FSL D4-0014	10403S mutant; referred to here as 10403S (GlcNAc^−^); nonsense mutation in *LMRG_00541*; deficient of N-acetyl glucosamine in cell wall teichoic acids	Denes et al., 2015 [15]
FSL D4-0119	10403S mutant; referred to here as 10403S (Rha^−^); nonsense mutation in *LMRG_00542*; deficient of rhamnose in cell wall teichoic acids	Denes et al., 2015 [15]
UTK S1-0004	10403S mutant; referred to here as 10403S (m_acid-resistance); nonsense mutation in *LMRG_00278* (encodes acid-resistance family protein HdeD)	This study
UTK S1-0010	10403S mutant; referred to here as 10403S (m_foldase); nonsense mutation in *LMRG_01613* (encodes foldase PrsA precursor)	This study
***Listeria* Phage**		
LP-018	*Homburgvirus*	Vongkamjan et al., 2012 [22]
LP-026	*Homburgvirus*	Vongkamjan et al., 2012 [22]
LP-037	*Homburgvirus*	Vongkamjan et al., 2012 [22]
LP-110	*Homburgvirus*	Vongkamjan et al., 2012 [22]
LP-114	*Homburgvirus*	Vongkamjan et al., 2012 [22]
LP-048	*Pecentumvirus*	Vongkamjan et al., 2012; Denes et al., 2014 [22,23]
LP-125	*Pecentumvirus*	Vongkamjan et al., 2012; Denes et al., 2014 [22,23]

**Table 2:** Average nucleotide identity (across aligned nucleotide percentage) for LP-018 and other homburgviruses as calculated by JSpeciesWS average nucleotide identity MUMer (ANIm).

	LP-026	LP-037	LP-110	LP-114	P70 ^a^	LP-018
**LP-026**	*	97.60 (93.40)	96.63 (93.26)	96.38 (93.62)	96.29 (91.95)	97.59 (93.72)
**LP-037**	97.60 (96.74)	*	96.83 (96.56)	97.49 (95.52)	96.29 (95.73)	97.71 (97.50)
**LP-110**	96.63 (95.96)	96.83 (95.92)	*	97.34 (93.17)	96.68 (95.42)	97.45 (95.62)
**LP-114**	96.38 (94.17)	97.49 (92.89)	97.34 (91.08)	*	96.31 (93.88)	97.41 (92.41)
**P70 ^a^**	96.29 (92.05)	96.29 (92.46)	96.68 (92.75)	96.31 (93.11)	*	96.31 (91.47)
**LP-018**	97.59 (96.21)	97.71 (96.74)	97.45 (95.37)	97.41 (94.31)	96.33 (93.90)	*

^a^ Type species of *Homburgvirus* genus.

**Table 3:** Mean efficiencies of plaquing of *Listeria* phages against phage-resistant mutants. Values represent the mean titer (*n* = 3) of each phage on each bacterial strain compared to the titer on the propagation host (*L. monocytogenes* strain MACK). Standard deviations are shown in parentheses.

Phage	*Listeria monocytogenes* Strain				
	10403S	FSL D4-0014	FSL D4-0119	UTK-S1-0004	UTK-S1-0010
		10403S (GlcNAc^−^)	10403S (Rha^−^)	10403S (m_acid-resistance)	10403S (m_foldase)
**LP-018**	1.3 (0.36)	0.0016 (0.0013)	0.061 (0.018)	0	0
**LP-018_m1**	1.5 (0.64)	0	0.13 (0.064)	0	0
**LP-018_m2**	0.91 (0.19)	0.49 (0.13)	0	0	0
**LP-048**	0.70 (0.11)	0.55 (0.21)	0	0.35 (0.091)	0.35 (0.24)
**LP-125**	0.80 (0.34)	0	0	0.76 (0.52)	0.78 (0.45)

## Supplementary Materials

The following are available online at https://www.mdpi.com/1999-4915/11/12/1166/s1, Figure S1: Growth curve of 10403S, 10403S (m_acid-resistance) and 10403S (m_foldase) at 25 °C. LB-MOPS was inoculated 1:100 with an overnight culture, grown to an OD_600_ of 0.1, then diluted 1:100 and measured by (A) CFU mL^−1^ or (B) OD_600_ for 12 h (data for A and B were collected from the same experiment). (C) Growth curve of 10403S and LP-018 resistant 10403S mutants at 25 °C. LB-MOPS was inoculated 1:100 with an overnight culture, grown to an OD_600_ of 0.1, then diluted 1:100 and measured by OD_600_ for 12 h. Data are mean values of three biological replicates, and error bars represent standard error. Table S1: Additional *Listeria monocytogenes* phage-resistant mutants isolated from this study.
