# Supplementary material for: Homburgvirus LP-018 Has a Unique Ability to Infect Phage-Resistant Listeria monocytogenes
Source: Viruses. 2019 Dec 17;11(12):1166. doi: 10.3390/v11121166 (PMC6950383; doi:10.3390/v11121166)
Supplement: Supplementary file 1 [file viruses-11-01166-s001.pdf]

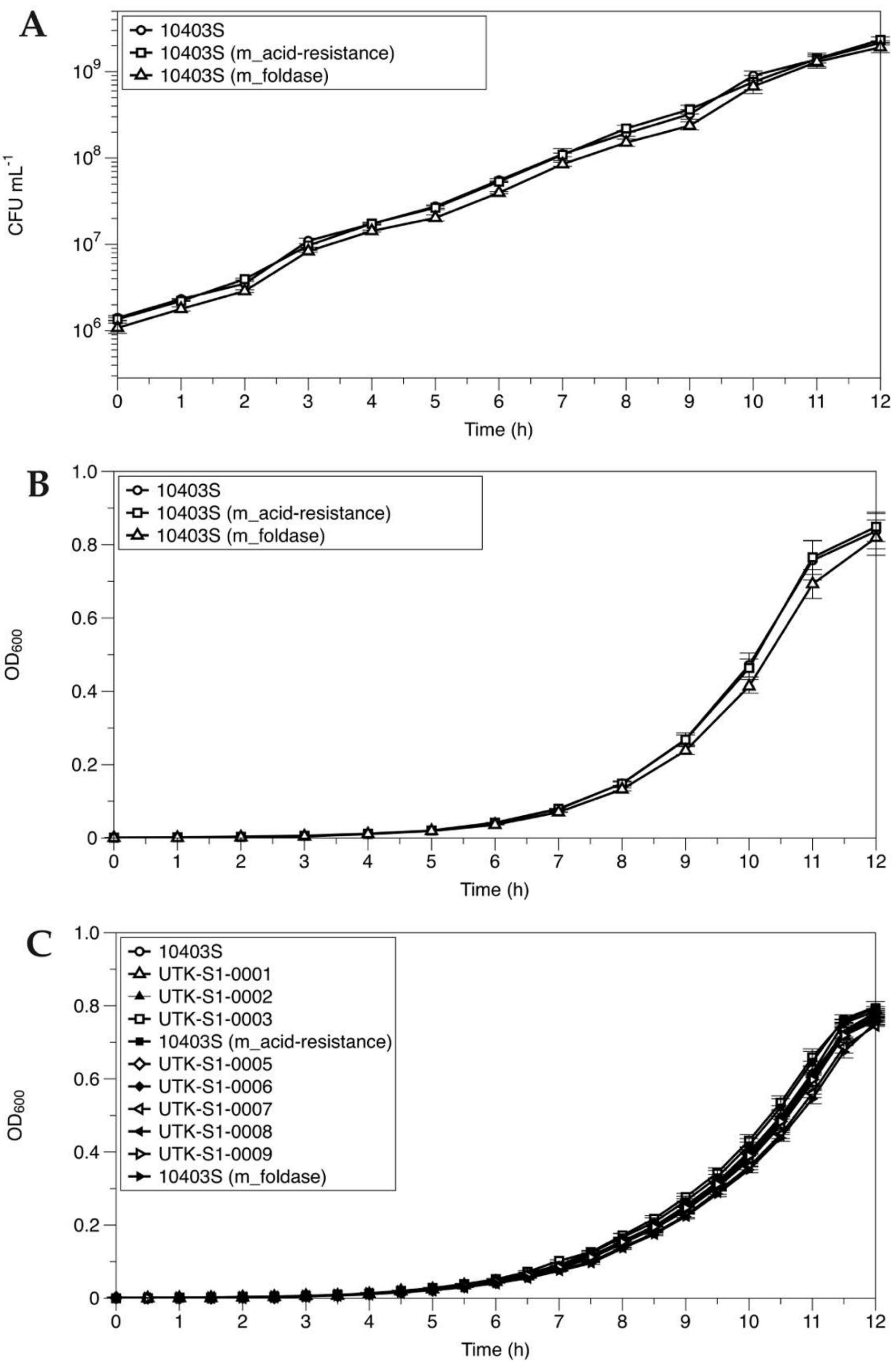

Supplemental Figure S1. Growth curve of 10403S, 10403S (m_acid-resistance) and 10403S (m_foldase) at 25°C. LB-MOPS was inoculated 1:100 with an overnight culture, grown to an OD_{600} of 0.1 then diluted 1:100 and measured by **(A)** CFU mL^{-1} or **(B)** OD_{600} for 12 hours (data for A and B were collected from the same experiment). **(C)** Growth curve of 10403S and LP-018 resistant 10403S mutants at 25°C. LB-MOPS was inoculated 1:100 with an overnight culture, grown to an OD_{600} of 0.1 then diluted 1:100 and measured by OD_{600} for 12 hours. Data are mean values of three biological replicates and error bars represent standard error.

Supplemental Table S1. Additional phage-resistant mutant strains of *Listeria monocytogenes*

Strain*	Description
***Listeria monocytogenes* mutant strains**	
UTK S1-0001	10403S mutant; frameshift mutation in *LMRG_01613* (encodes foldase PrsA2 precursor)
UTK S1-0002	10403S mutant; frameshift mutation in *LMRG_01613* (encodes foldase PrsA2 precursor)
UTK S1-0003	10403S mutant; nonsense mutation in *LMRG_00278* (encodes acid-resistance family protein HdeD), missense mutation in *LMRG_01441* (encodes a preprotein translocase subunit YajC)
UTK S1-0005	10403S mutant; frameshift mutation in *LMRG_00278* (encodes acid-resistance family protein HdeD)
UTK S1-0006	10403S mutant; frameshift mutation in *LMRG_00278* (encodes acid-resistance family protein HdeD)
UTK S1-0007	10403S mutant; nonsense mutation in *LMRG_01613* (encodes foldase PrsA2 precursor)
UTK S1-0008	10403S mutant; frameshift mutation in *LMRG_01613* (encodes foldase PrsA2 precursor)
UTK S1-0009	10403S mutant; nonsense mutation in *LMRG_01613* (encodes foldase PrsA2 precursor)

*All strains sourced from this study
